# Supplementary material for: Content-rich biological network constructed by mining PubMed abstracts
Source: BMC Bioinformatics. 2004 Oct 8;5:147. doi: 10.1186/1471-2105-5-147 (PMC528731; doi:10.1186/1471-2105-5-147)
Supplement: Additional File 2 — The original results of the above study (non-essential files are deleted to keep the file size under the limit set by BMC bioinformatics). [file 1471-2105-5-147-S2.bz2 › chilibotAdditionalFile2/dip05/313ID10606357E1370/html/S100A8_S100A9.html]

 


 **S100A8** and **S100A9** 
  
Found 177 abstracts in PubMed, retrieved 05.  
 

 What does Google say? 
 PDF only 
| .edu only 

---

**Interactive relationship** (e.g. stimulation, inhibition, etc)

**Stimulatory relationship**- An abundant 32kDa protein capable of stimulating IL 8 production was isolated from the secretion and identified as neutrophil cytoplasmic protein MRP 14  [ **S100A9** ] , which is the heavy polypeptide chain in the MRP 8  [ **S100A8** ]  14 heterodimer.  Ref: 12748056 Am J Respir Cell Mol Biol< MedlineTA>Am J Respir Cell Mol Biol,
**Neutral relationship**

**Non-interactive relationship** (e.g. studied together, co-existance, homology, etc.)

- MRP8  [ **S100A8** ]  MRP14  [ **S100A9** ]  levels in serum correlated with systemic parameters of disease activity erythrocyte sedimentation rate ESR r = 0.55, P = 0.005.  Ref: 12794836 Arthritis Rheum, 2003
- In contrast, MRP8  [ **S100A8** ] , MRP14  [ **S100A9** ] , and MRP8  [ **S100A8** ]  MRP14  [ **S100A9** ]  expression in the SLL of PsA patients is increased, particularly in perivascular regions, compared with that in RA and SpA patients, suggesting a central role of MRP proteins in transendothelial migration of leukocytes in PsA.  Ref: 12794836 Arthritis Rheum, 2003
- Calprotectin  **S100A8**   **S100A9** , an Inflammatory Protein Complex from Neutrophils with a Broad Apoptosis Inducing Activity.  Ref: 12808281 Biol Pharm Bull, 2003
- In addition, it was verified by immunohistochemical analysis that the expressions of Calgranulin A  [ **S100A8** ] , Calgranulin B  [ **S100A9** ] , and Cathepsin D were mainly located in cholesteatoma epithelium.  Ref: 12792315 Laryngoscope, 2003
- OBJECTIVE To analyze S 100 protein expression, in the form of myeloid related protein 8 MRP8  [ **S100A8** ] , MRP14  [ **S100A9** ] , and the heterodimer MRP8  [ **S100A8** ]  MRP14  [ **S100A9** ] , in psoriatic arthritis PsA patients compared with rheumatoid arthritis RA and spondylarthropathy SpA patients, and to determine the effect of methotrexate MTX on the MRP antigen expression in PsA patients.  Ref: 12794836 Arthritis Rheum, 2003
- Concentrations of MRP8  [ **S100A8** ]  MRP14  [ **S100A9** ]  in serum and SF were measured by enzyme linked immunosorbent assay.  Ref: 12794836 Arthritis Rheum, 2003
- Expression of MRP8  [ **S100A8** ] , MRP14  [ **S100A9** ] , and MRP8  [ **S100A8** ]  MRP14  [ **S100A9** ]  in synovium was determined by immunohistochemistry.  Ref: 12794836 Arthritis Rheum, 2003
- These included genes involved in cell proliferation and differentiation eg, calgranulin A  [ **S100A8** ] , calgranulin B  [ **S100A9** ] , psoriasin, thymosin beta 10 and cell invasion eg, cathepsin C, cathepsin D, cathepsin H.  Ref: 12792315 Laryngoscope, 2003
- RESULTS MRP8  [ **S100A8** ] , MRP14  [ **S100A9** ] , and MRP8  [ **S100A8** ]  MRP14  [ **S100A9** ]  levels were increased in serum, SF, and synovium from PsA, RA, and SpA patients.  Ref: 12794836 Arthritis Rheum, 2003
- Analyses by means of reverse transcription polymerase chain reaction showed enhanced expression of several genes including calgranulin A  [ **S100A8** ] , calgranulin B  [ **S100A9** ] , psoriasin, thymosin beta 10, cathepsin C, cathepsin D, and cathepsin H in cholesteatoma, supporting the findings from the gene array.  Ref: 12792315 Laryngoscope, 2003
- In all 3 groups, paired samples of serum and SF showed significantly higher MRP8  [ **S100A8** ]  MRP14  [ **S100A9** ]  levels in SF mean SD 1 10 1 99 ng ml median 1 00 than in serum 908 679 ng ml median 695 P = 0.0001.  Ref: 12794836 Arthritis Rheum, 2003
- Together with  **S100A8**  and  **S100A9** , it belongs to the calgranulin subfamily, i.e. it is mainly expressed in granulocytes, although there is an increasing body of evidence of expression in keratinocytes and psoriatic lesions.  Ref: 12777802 Acta Crystallogr D Biol Crystallogr, 2003
